# A267 RECENT IMMIGRANTS WITH INFLAMMATORY BOWEL DISEASE HAVE SIGNIFICANT HEALTH-CARE UTILIZATION FROM PRECONCEPTION TO POSTPARTUM: A POPULATION-BASED COHORT STUDY

**DOI:** 10.1093/jcag/gwad061.267

**Published:** 2024-02-14

**Authors:** P Tandon, V Huang, D Feig, R Saskin, C Maxwell, D Fell, C Seow, J Snelgrove, G Nguyen

**Affiliations:** University Health Network, Toronto, ON, Canada; Sinai Health, Toronto, ON, Canada; Sinai Health, Toronto, ON, Canada; Sunnybrook Health Sciences Centre, Toronto, ON, Canada; Sinai Health, Toronto, ON, Canada; Children's Hospital of Eastern Ontario, Ottawa, ON, Canada; University of Calgary, Calgary, AB, Canada; Sinai Health, Toronto, ON, Canada; Sinai Health, Toronto, ON, Canada

## Abstract

**Background:**

Previous studies have demonstrated an increase in health-care utilization, such as hospitalizations and emergency department (ED) visits, in immigrant people with IBD compared to non-immigrants. This may be even higher from preconception to postpartum due to heightened provider and patient concerns, though this remains to be confirmed.

**Aims:**

To characterize differences in health-care utilization from preconception to postpartum amongst immigrant and non-immigrant women with IBD.

**Methods:**

We accessed administrative databases to identify women (age 18-55) with IBD with a singleton pregnancy between 2003-2018. Immigration status was defined as recent (ampersand:003C 5 years of date of conception), remote (ampersand:003E5 years since date of conception), and none. Differences in ambulatory, ED, hospitalization and endoscopic visits during 12-months preconception, pregnancy and 12-months postpartum were characterized. World region of immigration origin was also ascertained. Adequate prenatal care was defined by using the validated R-GINDEX which assesses the date of first prenatal visit and total number of prenatal visits. Multivariable negative binomial regression was used to report adjusted incidence rate ratios (aIRR) and modified Poisson regression with quasi-likelihood models and robust error variance procedures were used to report adjusted relative risks (aRR) with 95% confidence intervals (95% CI).

**Results:**

8880 pregnancies were included, 8304 in non-immigrants, 96 in recent immigrants, 480 in remote immigrants. Compared to non-immigrants, recent immigrants had the highest rates of IBD-specific ambulatory visits during preconception (aIRR 3.06, 95% CI, 1.93-4.85), pregnancy (aIRR 2.15, 95% CI, 1.35-3.42), and postpartum (aIRR 2.21, 1.37-3.57) and the greatest rates of endoscopy visits during preconception (aIRR 2.69, 95% CI, 1.64-4.41) and postpartum (aIRR 2.01, 95% CI, 1.09-3.70). There were no differences in ED and hospitalization visits between groups. Compared to non-immigrants, immigrant women were much less likely to have a first trimester prenatal visit (remote: aRR 0.78, 95% CI, 0.65-0.93; recent: aRR 0.61, 95% CI, 0.42-0.88) or receive adequate prenatal care (remote: aRR 0.67, 95% CI, 0.46-0.98; recent: aRR 0.46, 95% CI, 0.22-0.96). Those immigrating from the Americas, Africa, or the Middle East were the most likely to be hospitalized during pregnancy, have a postpartum endoscopy visit, and receive inadequate prenatal care.

**Conclusions:**

There remain significant disparities in health-care utilization amongst immigrant women with IBD from preconception to postpartum. Health policy endeavors are required to provide standardized and equitable care to all women with IBD during these otherwise high risk periods.

**Funding Agencies:**

CCC, CIHR

